# Behavioral and Physiological Responses of Calves to Marshalling and Roping in a Simulated Rodeo Event

**DOI:** 10.3390/ani6050030

**Published:** 2016-04-28

**Authors:** Michelle Sinclair, Tamara Keeley, Anne-Cecile Lefebvre, Clive J. C. Phillips

**Affiliations:** 1 Centre for Animal Welfare and Ethics, School of Veterinary Sciences, The University of Queensland, Gatton, Queensland 4343, Australia; m.sinclair6@uq.edu.au (M.S.); a.lefebvre_12@envt.fr (A.-C.L.); 2 School of Agriculture and Food Sciences, The University of Queensland, Gatton, Queensland 4343, Australia; t.keeley@uq.edu.au; 3 École Nationale Vétérinaire de Toulouse, Toulouse University, Toulouse 3115, France

**Keywords:** animal welfare, calf, cattle, rodeo, roping

## Abstract

**Simple Summary:**

Rodeos often include a calf roping event, where calves are first lassoed by a rider on a horse, who then dismounts, ties the calves’ legs, lifts it from the ground and releases it back to the floor. We tested whether calves that were familiar to the roping experience stress during the roping event, and found increased concentrations of stress hormones in their blood after the roping. We also found increased concentrations of stress hormones in the blood of calves that had never been roped before but were just marshelled across the arena by the horse and rider. We conclude that the roping event in rodeos is stressful for both experienced and naïve calves.

**Abstract:**

Rodeos are public events at which stockpeople face tests of their ability to manage cattle and horses, some of which relate directly to rangeland cattle husbandry. One of these is calf roping, in which a calf released from a chute is pursued by a horse and rider, who lassoes, lifts and drops the calf to the ground and finally ties it around the legs. Measurements were made of behavior and stress responses of ten rodeo-naïve calves marshalled by a horse and rider, and ten rodeo-experienced calves that were roped. Naïve calves marshalled by a horse and rider traversed the arena slowly, whereas rodeo-experienced calves ran rapidly until roped. Each activity was repeated once after two hours. Blood samples taken before and after each activity demonstrated increased cortisol, epinephrine and nor-epinephrine in both groups. However, there was no evidence of a continued increase in stress hormones in either group by the start of the repeated activity, suggesting that the elevated stress hormones were not a response to a prolonged effect of the initial blood sampling. It is concluded that both the marshalling of calves naïve to the roping chute by stockpeople and the roping and dropping of experienced calves are stressful in a simulated rodeo calf roping event.

## 1. Introduction

Organised rodeos began as competitive events to increase and display practical stockperson skills required for cattle ranching [1]. Roping, riding and wrestling cattle to the ground to brand or provide medical treatment were operational skills required in ranching, with strength and speed particularly valued [2]. The earliest recorded rodeo event was at Deer Trail in Colorado in the 1860s [3], and the first recorded and organized event in Australia was held in 1888, when the National Agricultural Society of Victoria hosted a “rough riding” competition at their annual show [4]. As a nation with a major cattle industry, rodeos were popular in Australia, with a 1927 South Australian rodeo attracting an estimated 50,000 spectators [5]. Recently, the Houston Rodeo in Texas attracted a record 2,485,721 people [6], and the Calgary Stampede in Canada attracts over 1,000,000 people each year [7]. The number of non-human animals engaged in rodeo activities is unknown, however the Australian Pro Rodeo Association (APRA) estimates “30,000 usages of animals” over a 15 year period at APRA-affiliated rodeos [8]. Of these, 821 per year were used in 2013/2014 for calf rope and tie events (Karen Burraston, APRA, personal communication). These figures do not include animals used in rodeos managed by other rodeo groups in Australia, such as the National Rodeo Council of Australia (NRCR) or rodeos not run by a professional association.

Although cattle ranching is evolving from being horseback-based to vehicle-based, rodeos showcasing horseback-based cattle ranching skills remain of interest for many, especially in predominately rural areas. However, there are significant concerns for the welfare of both the cattle and horses involved in rodeos. As early as 1924, Lord Lonsdale, president of the Olympia circus, stopped public displays of steer roping in the circus, based on his concern for the wellbeing of the animals [9]. Calf roping is a particularly contentious rodeo activity. It is a timed event involving the release of a calf from a holding pen, through a crush chute into an arena to be chased by a competitor on horseback, who attempts to lasso the calf while both are moving. Once the calf is captured *via* lasso, the rope tightens around the calf’s neck and forces it to a halt. The competitor dismounts, lifts the calf and drops it onto its side, in a position of lateral recumbency. It is then restrained by the competitor, who ties three legs together and raises his hands to signify completion of the task. The event usually lasts 7–10 s when performed by champion level competitors, with the current record standing at 6.3 s in Canada [10]. Although many believe that calf roping is cruel [11,12,13,14]; there is little published evidence of suffering in the calves. Webster writes that calves are “isolated, forcibly thrown down and trussed up….in an aversive experience [14]”. Some authors quote injury rates as evidence of the negative experiences of calves [15,16], but these are challenged by others [17,18]. Philosopher Bernard Rollin raises ethical concerns with entertainment that involves calves suffering stress and pain [13].

There have been attempts recently to regulate the handling of calves in this event: draft Australian Animal Welfare Standards and Guidelines for Cattle [19] require that a person handling cattle must not: (1) lift cattle off the ground by only the head, ears, horns, neck or tail unless in an emergency; or (2) drop cattle except to land and stand on their feet.

Comprehensive evaluation of the effects of calf roping on calf welfare requires assessment of both calf behavior and physiology. Cortisol is a glucocorticoid hormone released into the blood by the adrenal cortex in times of stress resulting in an increase in blood sugar through gluconeogenesis to aid in the rapid metabolism of carbohydrate, fat and protein, nutrients needed in stressful situations [20,21]. Complimentary to the longer lasting effects of cortisol release, epinephrine (adrenaline) and nor-epinephrine (noradrenaline) are empirically-supported measures of acute short term stress. Secreted by the adrenal glands, epinephrine is a rapid release hormone that serves to dilate the coronary artery, induce tachycardia, increase muscle strength, and enhance sugar metabolism and blood pressure, preparing the body for an immediate “fight or flight response” [22]. Nor-epinephrine has a similar endocrinological response and excretory route to the related epinephrine, but is more correlated to physical stimuli, whereas epinephrine is more related to psychological stimuli [23]. Epinephrine and nor-epinephrine blood serum analysis has been used to effectively measure acute bovine stress levels, for example, to indicate stress at slaughter [24].

Stress levels of calves have been reported in one study to be unaffected after being subjected to transportation, loading into chutes and roped, as determined from change in blood cortisol from before onset of the sequence of events to 10–30 min after the completion of all events [25]. This is in contrast to a study where cortisol concentrations of all calves, including control animals that remained at a farm, or were just transported were reported to be elevated above baseline levels [26], indicating an increase, but no significant variation according to treatment. In this later study, however, variable periods of 185 to 295 min elapsed from initial to final blood sampling which may have allowed extinction of the stress response [26], and with both studies the activity of roping, separate from transportation, was not assessed. The diurnal rhythms of cortisol in bovine plasma may also affect the measured cortisol levels [27,28,29]. Cortisol levels have been reported to increase from approximately 22 to 44 ng/mL within 24 h of calf roping, an increase in creatinine kinase for at least 24 h after calf roping was also reported [30]. In addition, sham chewing, head shaking and coughing was observed shortly after the roping, over a period of about 10 s. After sudden cessation of locomotion, some calves were observed to be hesitant or slow to get to their feet, appearing disorientated or dizzy, or walked before running away from the site [30].

The current study aimed to determine whether rodeo calf roping is stressful to the animals by assessing the behavior and physiology of calves before and after two major components of the roping event. First, we used calves that were naïve to the roping event to examine the effects of marshalling them on horseback across the roping arena. Second, we examined the effects of the full roping procedure on calves that had experienced it before. We hypothesized that both marshalling of naïve calves and roping of experienced calves would lead to an increase in both the acute stress hormones, epinephrine and nor-epinephrine, and the longer-term stress hormone, cortisol. Furthermore we hypothesized that calves would not habituate to the roping procedures. To test this hypothesis the effects of roping on calves that had been roped previously were measured to assess if they showed a stress response despite previous experience of being roped.

## 2. Materials and Methods

Animal Ethics approval was granted for these procedures by The Department of Agriculture, Forestry and Fisheries in Queensland, Australia (CA 2014/04/757). Calf roping was performed in accordance with the standards of the Australian Professional Rodeo Association [31].

### 2.1. Test Arena

**Figure 1:**
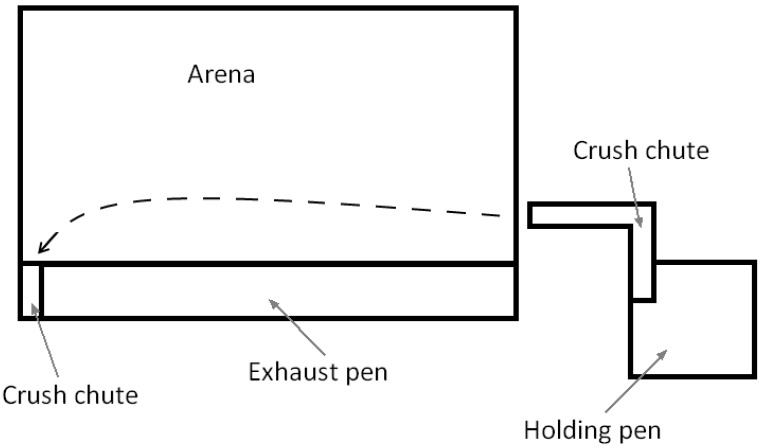
Test facility, with holding pen, crush chutes and exhaust pen for return to holding pen or paddock, together with the route taken by Naïve Marshalled (NM) calves.

The facility was located at Destiny Downs (Emerald, Queensland, Australia) and included a 90 by 55 m rodeo training arena with leveled sand substrate (Figure 1). A holding pen was located at one end, joined to a chute for leading calves individually out of the pen to ready them for release into the arena. At the opposite end of the arena was another crush and gate through which calves could exit the arena via an exhaust pen. This followed a fenced path back alongside the arena into the holding pen or a paddock. The facility design allowed for 3–5 calves to be in the chute at any time, with the calf at the front moved into a crush that could be manually closed behind it to prevent it backing out or other calves entering the arena when the crush was opened. On release of the crush the calf was free to move forward into the arena.

### 2.2. Study Animals and Conditions

Twenty weaned four to eight months old Longhorn cross Droughtmaster calves were studied: twelve heifers and eight steers, with a mean weight of 101.3 kg (SEM 2.07). The calves were bred and resided at Destiny Downs for the purpose of rodeo performance and eventual slaughter. All calves were reared with their dams until the point of weaning, and were mustered with horses, dogs and motorbikes one month before the commencement of the study. On the holding property after mustering, the calves had visual contact with humans during feed time as grain bins and hay feeders were replenished. Human contact prior to being exposed to the test arena was consistent across groups and individual calves, and is representative of the common practice for rodeo animals on farm for the National Rodeo Association.

The diet of all calves consisted of calf weaner pellets (Blue Ribbon Stockfeeds, 18% crude protein), buffel grass and lucerne hay, and all were in good health. The experimental procedure was carried out at Destiny Downs; after the stualsdy the calves were returned to paddocks on this property with the rest of the herd.

After morning feeding on the day of study, the calves were separated into two groups (treatments Naïve Marshalled and Experienced Roping) in holding pens adjoined to the arena. The weather conditions on the day was moderate, fine and clear, with temperature ranging from 15–18 °C and humidity of 28%–39% during the hours of testing.

### 2.3. Experimental Procedure

During the study each group was exposed to activities (either marshalling or roping), and blood sampling was conducted by an experienced veterinarian immediately before and after these activities. In the case of the initial blood sampling, the veterinarian operated outside of the chute, and there was no behavioural response (flinch, kick or step) to venepuncture. In the case of the post-treatment blood sampling the calves were restrained, as normal at the end of the roping event. The procedure was repeated after a 2 h rest period for each group. The 2 h time period was imposed to allow rest time for the calves, similar to the minimum rest time afforded in a live rodeo setting.

#### 2.3.1. Group NM—Naïve Marshalled

Group Naïve Marshalled (NM, 10 calves) were randomly selected from the on-property calves that had not previously been exposed to the holding pen and chute, and only once to the arena. NM calves had also never been previously exposed to rodeo roping and were not exposed in this study. To mitigate against possible neophobic responses, the naïve marshalled calves were previously exposed to being marshalled across the arena by a horse and rider 5 times one week before the commencement of the study, as is on-farm routine in preparation for the commencement of roping training. However, the chute and the holding pen were novel to these calves. 

The holding pen was configured to allow three calves into the chute leading to the crush, and the calf at the front into the crush. At this time the calf was weighed on a platform with an inbuilt scale and blood was collected by jugular venepuncture into a 5 mL Vacutainer with 18 gauge needle. On release from the crush into the arena the calf was allowed to make its own way to the gate at the far end of the arena, passively marshaled by a professional champion calf roping competitor on horseback at a distance of approximately 20 m. Two researchers followed a further 10 m behind to take blood samples and video-record behavior. If the calf veered away from the intended path towards and into the gated crush at the end of the area, the stockperson mustered the calf back. On entry to the second chute the calf was restrained and jugular venepuncture was again performed, with blood collected into a 5 mL Vacutainer using an 18 gauge needle.

#### 2.3.2. Group ER—Experienced Roping

Group Exposed Roped (ER, 10 calves) were randomly selected from the Destiny Downs calves that had been regularly exposed to the arena, had previously experienced rodeo training at approximately 10 off-site rodeo ropings within the two months prior to the events described here. They were roped in this study in an attempt to simulate as closely as possible a normal rodeo event, but without the vehicle transportation and distraction of crowds of people and music.

The holding pen with the calves in Group ER was configured as for Group NM, allowing three calves into the chute leading to the crush, and the calf at the front into the crush for weighing and blood sampling. On a cue from the same professional champion calf roping competitor on horseback who was waiting in the arena, the calf was released from the crush. During the traverse of the arena, the calf was chased at high speed on horseback, roped and secured with a lasso, which was fitted with a “Ropersmate” rope pulley device designed to reduce the jerk impact on calves immediately after capture around the neck. The competitor then dismounted from the horse and approached the calf, lifted it from the ground to hip height and dropped it to the ground into a lateral recumbent position. The competitor then tied three of the calf’s legs together and raised both hands to signal that the activity had been completed. While the calf was on the ground jugular venepuncture was again performed by two researchers, and blood collected into a 5 mL Vacutainer using an 18 gauge needle. The calves were then promptly untied and allowed to move towards the end of the arena and out of the gate without human assistance. A third blood sample was planned for both groups exactly 15 min after the start of the procedure, but had to be abandoned on the day of the study because of concerns about venous integrity and the welfare of the calves. The lasso missed one ER calf during roping and the calf was returned to the handling pen and underwent the procedure again.

All events and time points were carried out on one day, with two replicates of the treatment. Blood samples were stored in a cold box and allowed to coagulate, but not frozen to prevent degradation of sample through haemolysis. Samples were transported with the researchers by air to the laboratory on the same day and refrigerated to 4 °C. On return to the laboratory all samples were immediately centrifuged (Beckman Coulter, J6-M1, Yeerongpilly, Queensland, Australia) at 3100× *g* for 10 min to separate serum from cells, and serum was extracted using pipettes into aliquot tubes and then frozen at −28 °C before analysis for cortisol, epinephrine and nor-epinephrine concentrations.

### 2.4. Stress Hormone Analysis

Serum cortisol concentration was determined by a cortisol enzyme-immunoassay [32]. Serum samples were diluted 1:6 in assay buffer prior to analysis and a serial dilution of a pool of randomly-selected serum samples demonstrated parallelism with the standard curve. The cortisol antibody (R4866, C.J. Munro, University of California, Davis, CA, USA) cross-reacts with cortisol 100%, prednisolone 9.9%, prednisone 6.3%, cortisone 5% and <1% with corticosterone, esoxycorticosterone, 21-desoxycortisone, testosterone, androstenedione, androsterone, and 11-desoxycortisol [33]. Intra-assay and inter-assay coefficients of variation were 4.34% and 7.92% respectively.

Serum epinephrine and norepinephrine concentrations were determined by enzyme-immunoassay (EIA) as described by the manufacturer (Abnova; Product KA1877, Taipei, Taiwan). The control values of the Abnova EIAs were within the reported range. Intra-assay and inter-assay coefficients of variation for epinephrine were 5.18% and 4.79% and for norepinephrine were 4.80% and 2.37% respectively.

Serum cortisol, epinephrine and norepinephrine values are expressed as ng/mL. The absorbance values of all enzyme-immunoassays were determined using an Epoch microplate reader and Gen5 software (Biotek) (BioTek, Winooski, VT, USA). A test and reference filter of 405 and 540 was used for the cortisol EIA and 450 and 630 for the epinephrine and norepinephrine EIAs.

### 2.5. Behavioral Analysis

Calf behavior was recorded continuously in real time by video camera (Kobi CCD Video Camera, Model K-32HCVF, Ashmore, Queensland, Australia) during exposure to each activity. A digital video recorder (Kobi H.266, Model XQ-L 900H, Ashmore, Queensland, Australia) was used to record the images, and the video data were then analysed using a continuous recording of each animal and Cowlog 2.0 behavior software (Research Centre for Animal Welfare, University of Helsinki, Helsinki, Finland) for coding of behaviors [34]. Exit times from the chute were recorded for calves in each group. The duration of time spent by NM calves in the following mutually exclusive states was continuously recorded: running, trotting and walking, together with distance travelled. Similarly, the duration of time spent by ER calves was continuously recorded for behaviors in the ethogram developed for this activity, except that eye roll and vocalizations were noted as one:zero occurrences on the day (Table 1).

### 2.6. Statistical Analysis

Behavior and endocrinological parameters were analysed using a general linear model with the following factors: treatment group, calf, replicate and before/after treatment, together with the respective two-way interactions between these factors, using the statistical package Minitab 16 (Minitab Inc., State College, PA, USA). During analyses, all data were checked for normal distribution of residuals using the Anderson-Darling test. For data not satisfying the Anderson-Darling test, log_10_ transformations were made and back-transformed means are reported in addition to transformed data. The log_10_ parameters produced a normal distribution of residuals.

## 3. Results

### 3.1. Physiological Responses

Cortisol, epinephrine and nor-epinephrine concentrations were all increased after the activity, compared with before, in both NM and ER calves (Table 2). The cortisol and nor-epinephrine concentrations were greater in NM than ER calves, but for epinephrine there was no difference between treatments. There were no significant differences (*p* < 0.05) between replicate 1 and 2 and no significant interactions between NM/ER activity and before/after values. 

### 3.2. Behavior

Calf speed during treatment was greater for ER (3.05 m/s) than NM calves (2.29 m/s) (SED 0.160, *p* < 0.001). Mean speeds for replicates 1 (2.58 m/s) and 2 (2.76 m/s) did not differ between treatments (SED 0.160, *p* = 0.16). The mean total time for the run (Start running—Stop running, Table 1) was longer for NM calves (39.3 s) than ER calves (3.5 s). On average, NM calves spent most time trotting (51.0%), then running (36.4%), and least time walking (11.7%) (SED 11.42, *p* = 0.005). All ER calves ran 100% of the time and their speed of running was faster (3.03 m/s) than NM calves (2.70 m/s) (log_10_ m/s 0.48 and 0.43, respectively, SED = 0.021, *p* = 0.009). There were no differences between replicates one and two in the % time spent running, trotting or walking, or in the speed of the calves in these different gaits (Table 3 and Table 4). One calf was roped around the leg rather than the neck and was observed to vocalize repeatedly until subsequent release. Apart from this, no ER or NM calves vocalized at any time following release from the chute. Following the calf drop all ER calves displayed eye roll while laterally recumbent following roping, but not during chute restraint, and none of the NM calves displayed this during sampling before or after traversing the arena.

For the ER calves, mean time from the exit from the crush to (a) the settling of the lasso on the neck was 1.77 s SEM 0.092; (b) the end of the run was 3.05 s SEM 0.171; (c) the lasso being tight around the neck 3.11 s SEM 0.194; (d) the lifting of the calf 6.36 s SEM 0.473. The calf was then dropped and took 0.33 s, SEM 0.034, to hit the floor. The time taken from the start of tying the knot to the end, signified by raising his hand to the air was a further 2.60 s, SEM 0.241. There was a tendency for the cowboy to tie the knot faster the second time (2.20 *vs.* 2.99 s, SED 0.76, *p* = 0.077). Mean time from passing the lasso around the legs to the raising of the hand was 3.76 s, SEM 0.334. Apart from tying the knot there were no significant differences between the times for the first and second repeat (Table 3).

## 4. Discussion

Both groups demonstrated an increase in neuroendocrine stress responses as a result of treatment. Jugular venepuncture may be a cause of stress to cattle [35], but continued elevation of the hormone parameters following initial sampling is unlikely to explain the increase as a result of treatment, because stress hormones were not increased at the start of the second replicate, compared with the first. Continued elevation of the hormone parameters following initial sampling as the reason for the increase in stress hormones is unlikely to explain the increase as a result of treatment because stress hormones were not increased at the start of the second replicate, compared with the first. The increase in cortisol for NM calves compared with ER calves could have been influenced by the time that had elapsed before post-treatment sampling began: mean 39 s for treatment NM and 9.3 s for treatment ER. However, cortisol takes up to 15–20 min to respond to a chronic stressor, and it is likely that peak values would not have been reached until after either activity was completed [26]. It is most likely that NM calves found the handling more stressful than the ER calves found handling and roping, with which they were familiar. Repeated handling can attenuate cortisol responses in a chute [35], but the experience of NM calves in being marshalled across the arena probably did not generalize to their experiences in the chute. Animal learning is very specific, for example training a horse to tolerate a blue and white umbrella did not transfer to an orange tarpaulin [36]. This suggests novelty played an important role in the stress response of the calves. Increased cortisol has been documented in physical exertion in humans [37], although this has not been specifically studied in bovines. If applicable, the increase in cortisol here may also be partly attributed to the neuroendocrine response to physical exertion, as NM calves had a longer distance to run. The absence of any difference in epinephrine concentrations in NM and ER calves, however, indicates that psychological stress, as demonstrated by increased nor-epinephrine, played a significant role in both NM and ER calves.

In contrast to research by Ferguson *et al.* (2013), this study found a marked increase in cortisol after roping [25]. Baseline cortisol levels of 32–61 ng/mL in Ferguson *et al*. study were greater than baseline levels reported elsewhere, which is discussed below [25]. As with the blood cortisol response, in our study epinephrine and nor-epinephrine increased after ER and NM treatments, signifying an acute stress response [37]. No difference was found between the first and second replicates, indicating a lack of habituation to the aversive stimuli, as previously described by Grandin and Shivley [38].

Some stress is normal and within non-damaging limits, therefore it is important to compare these study results to empirically-supported basal levels. Grandin (1997) suggests that baseline cortisol levels in calves are from 3–6 ng/mL and a value above 70 ng/mL may indicate rough handling [26]. However, we cannot be sure that this is a critical level that applies in this instance as the calf genotype and age, type of handling, environment *etc.* all differ between studies. Thus, even though cortisol was increased by both treatments, and levels remained below the critical level identified by Grandin, it is still possible that the procedures caused damaging stress, which we consider to be that which adversely affects the animal’s welfare. A level of 20 ng/mL in the NM calves indicates elevation above baseline, and furthermore it was not possible to distinguish between the stresses involved in marshalling the naïve calves prior to the first blood sample and the stress involved in marshalling them across the arena.

Maintenance of ER calves close to baseline suggests that the calves did not anticipate the stress of roping sufficiently far in advance for cortisol to become elevated. However, even if anticipation of roping did not significantly elevate cortisol, the faster speed of ER calves across the arena suggests that a vigorous escape attempt occurred. The elevated epinephrine relative to nor-epinephrine following this activity suggests a psychological response. Ratios after treatment were 8.2:1 for ER calves but only 2.4: 1 for NM calves. The consistent eye white response (100% of calves) at the point of lift and drop during the rope tying may also be indicative of significant stress, since eye white responses in dairy cows indicate stress [39,40,41]. These eye roll responses may indicate that the animal is blocking out its environment from view. Vocalisation was confined to one ER calf that was roped around the leg not the neck, possibly because tightening the noose around the neck inhibits vocalisation. In NM calves, cortisol levels appear to have increased above baseline even before treatment was applied. This is likely to have been due to the placing of the calves into the handling pen, a procedure which they had not experienced before. However, the fact that cortisol was not elevated above 20 ng/mL at the start (or end) of the second traverse of the arena suggests that stress levels were not extreme.

The horse riders in this study were professional champion cowboys, and unlikely to be typical of general rodeo contestants. More junior cowboys not only rope and tie over a longer period, they may also have difficulty lifting the calf into a suitable position from which to drop it to the ground. The nature of this drop may be important if it stuns the calf into submission for the tying process. Eyewhite responses suggest that this may be the case. At rodeos new competitors can take over 20 s to effect the tie down roping, which would have a much greater impact on stress levels [42]. Proactive animal welfare efforts by APRA, such as the creation and implementation of the “Ropersmate” rope pulley device should be evaluated for any reduction in acute stress impact.

This study had limitations in that it was only possible to examine increases in stress response across two varied treatment groups. Although this study was initially planned to include a control group, rather than 2 varied treatment groups, on farm operation did not allow for this within this study. Therefore, this study is one of comparison between the naive marshalled (NM) calves and the experienced roped (ER) calves. Further insight and comparison may be gained by including a control group in later studies of experienced but only marshalled calves (EM), and another treatment of naïve and roped calves (NR) where operational practicalities allow.

In addition to this, cortisol reaches maximum levels in calves at 1 h, and these samples were taken earlier and more immediately. The cortisol responses and differences between treatment groups in the short time frame however is still indicative of a significant stress response, although maximal responses would not have been captured since blood was not taken an hour after the exposure. Cortisol responses in particular are difficult to compare between studies, as many variables influence concentrations. Further evaluation of maximal cortisol responses would be particularly beneficial. Some behavioral responses, in particular eyewhite, also warrant further exploration. 

Ultradian cortisol rhythms may have influenced the results. In this study an increase of 1.8 ng/mL over a mean 20 s period was observed, with the short term time frame making interference less likely. Other research is conflicting, with reports that ultradian rhythms in cattle have much longer periods of approximately 120 min [29] or that there are no rhythms [28]. The process of handling, and of taking blood can increase cortisol but this study focuses on the difference between treatment groups rather than simply the cortisol level.

The results of this study have a number of implications for animal welfare. Detection of an acute stress response in two components of calf roping, marshaling calves and roping of experienced calves, suggests that the experience is aversive. Further study would be needed to confirm this. APRA limits the number of times that calves can be utilized at a rodeo to three, but repeated exposure to acute stress in this way may be unacceptable from an animal welfare standpoint in the light of this evidence. Further study is necessary to confirm this. Women’s events do not include the calf drop, so this could be considered for adoption into men’s events also, as could further research into the difference in physiological and behavioural stress responses between women’s and men’s events.

## 5. Conclusions

Increases in blood cortisol, epinephrine and nor-epinephrine in both naïve calves marshaled across a roping arena indicate that the marshalling across the arena was at least initially stressful, notwithstanding the possibility that initial blood sampling caused prolonged elevation, discussed above. The repeat roping for the experienced roped calves on the day of the study also produced an acute stress response. The physiological evidence suggests that naïve calves marshalled across the roping arena were acutely stressed by the initial handling, with an adrenergic response, and there was also some evidence of the development of a more chronic stress response. Behavioral evidence suggests that experienced roped calves had a flight response to the presence of the pursuing rider and eye white responses may also indicate a stress response to roping. Physiological evidence was that they too experienced an adrenergic response to the roping and the start of a hypothalamopituitary axis response. Further research could examine the stress responses in calves that were jerked by the rope, which did not happen in this study. 

## Figures and Tables

**Table 1:** Behaviors recorded for calves in treatment group Experienced Roping (ER).

Behavior	Description
Start running	Calf leaves the crush chute
Stop running	Calf stops running at entry to second chute (NM) or arrested by lasso (ER)
Lasso around neck	Lasso passes over the head and settles around the neck of the calf
Lasso around leg	Lasso fails to pass the head and captures the back leg
Lasso tight	Lasso is tightened around the neck or the leg
Lift	Cowboy lifts the calf by two legs
Calf dropped	The calf is dropped by the cowboy
Tie leg	Cowboy passes the rope around the front leg of the calf
Start tying	Cowboy begins to form the rope tie around the leg
End tying	Cowboy finishes the rope tie and raises his hands to signal completion of the event
Vocalisation	Calf emits noise from mouth
Eye roll	Calf rolls eye to reveal over 50% eyewhite to pupil ratio.

**Table 2:** Least square mean concentrations of stress hormones before and after treatment in Naïve Marshalled calves (NM) and Experienced Roped (ER) calves. There were no significant (*p* < 0.05) two way interactions between the factors treatment, before/after and replicate.

	Replicate 1				Replicate 2				SED ^1^	Treatment *p* Value	Before/After *p* Value	Replicate *p* Value
	Treatment NM		Treatment ER		Treatment NM		Treatment ER					
	Before	After	Before	After	Before	After	Before	After				
Cortisol												
log_10_ ng/mL	1.19	1.28	0.50	0.75	1.14	1.32	0.47	0.69	0.127	<0.001	0.005	0.66
ng/mL	15.66	19.02	3.14	5.68	13.76	21.10	2.96	4.91				
Epinephrine												
log_10_ ng/mL	−0.57	−0.41	−0.48	−0.26	−0.38	−0.35	−0.46	−0.24	0.123	0.26	0.01	0.22
ng/mL	0.266	0.388	0.331	0.549	0.421	0.442	0.350	0.581				
Nor-epinephrine												
log_10_ ng/mL	−1.08	−0.81	−1.60	−1.26	−1.10	−0.69	−1.46	−1.07	0.192	<0.001	0.007	0.29
ng/mL	0.082	0.156	0.025	0.055	0.079	0.204	0.035	0.085				

^1^ Standard error of the difference between two treatments.

**Table 3:** Least square means for behaviours in replicates 1 and 2 for Naïve Marshalled (NM) calves.

	Replicate 1	Replicate 2	SED	*p* Value
Run, % time	28.9	43.8	2.02	0.14
Trot, % time	51.2	49.9	2.58	0.93
Walk, % time	19.1	4.3	2.10	0.17
Run speed, m/s	2.87	2.61	0.316	0.32
Trot speed, m/s	2.31	2.54	0.375	0.35
Walk speed, m/s	2.16	2.49	0.287	0.13

**Table 4:** Least square means for behaviours in replicates 1 and 2 for Experienced Roped (ER) calves.

	Replicate 1	Replicate 2	SED ^1^	*p* Value
Speed, m/s	3.06	3.05	0.760	0.15
Run time, s	3.48	3.51	0.329	0.54
Exit to lasso around neck, s	1.67	1.87	0.240	0.22
Exit to lasso tight, s	3.11	3.11	0.351	0.99
Exit to lift, s	7.03	5.71	0.547	0.12
Drop to floor, s	0.31	0.34	0.146	0.59
Tying knot to end, s	2.99	2.20	0.391	0.077
Lasso legs to end	4.16	3.36	0.461	0.18

^1^ Standard error of the difference between two treatments.
